# Séroprévalence et facteurs associés à l'hépatite virale B chez les gestantes à Parakou en République du Bénin

**DOI:** 10.11604/pamj.2019.33.226.19429

**Published:** 2019-07-18

**Authors:** Khadidjatou Saké Alassan, Rachidi Sidi Imorou, Honorat Sonombiti, Kabibou Salifou, Edgar-Marius Ouendo

**Affiliations:** 1 Service de Médecine Interne, CHUD-B, Parakou, République du Bénin; 2 Service de Gynécologie-Obstétrique, CHUD-B, Parakou, République du Bénin; 3 Institut Régional de Santé Publique, Ouidah, République du Bénin

**Keywords:** HBsAg, pregnant women, Parakou, AgHBs, gestantes, Parakou

## Abstract

L'objectif est de déterminer la séroprévalence et les facteurs associés à l'hépatite virale B chez les gestantes au Centre Hospitalier Universitaire Départemental du Borgou (CHUD-B). Il s'est agi d'une étude transversale à visée descriptive et analytique avec recueil prospectif des données. Elle a porté sur les gestantes suivies et/ou ayant accouchées au CHUD-B du 1^er^ avril 2017 au 30 juin 2017. Étaient incluses, celles ayant données leur consentement à participer à l'étude. Les variables étudiées étaient les données sociodémographiques des gestantes et le résultat de l'AgHBs. La recherche de l'AgHBs sur les prélèvements sanguins des gestantes était faite par un test biologique de diagnostic rapide. Les résultats positifs étaient confirmés par la technique ELISA. Deux cent quatorze (214) gestantes étaient incluses dans l'étude. Leur âge moyen était de 26,73 ± 5,68 ans. L'âge médian de la grossesse en cours était de 31 semaines d'aménorrhée (SA) avec des extrêmes de 4 SA et 42 SA. Parmi elles, 30 femmes étaient dépistées positives à l'AgHBs soit une prévalence de 14,02%. Les facteurs associés à ce portage étaient la pratique des scarifications, l'antécédent personnel d'ictère et les antécédents familiaux d'hépatite virale B. La séroprévalence de l'hépatite virale B chez les gestantes au CHUD-B est élevée ce qui rend bien compte du problème majeur de santé publique que pose l'infection par le virus de l'hépatite B sous nos cieux.

## Abstract (en)

The purpose of this study is to determine the seroprevalence and the factors associated with viral hepatitis B among pregnant women at the Departmental University Hospital of Borgou (CHUD-B).We conducted a cross-sectional descriptive and analytical study with prospective collection of data. It focused on pregnant women treated and/or having given birth at the CHUD-B from 1 April 2017 to 30 June 2017. The study included patients who gave their consent to participate in the study. The variables studied were the sociodemographic data of pregnant women and the result of HBsAg test. The detectionof HBsAg in blood sampling of pregnant women was performed using a biological test for rapid diagnosis. Positive results were confirmed by ELISA technique. Two hundred and fourteen (214) pregnant women were included in the study. The average age of patients was 26.73±5.68 years. The mean gestation age was 31 weeks of amenorrhea (WA) ranging from 4 WA and 42 WA. Among them, 30 women were positive for HBsAg, reflecting a prevalence of 14.02%. The factors associated with this colonization were the practice of scarification, a personal history of jaundice and family history of viral hepatitis B. The seroprevalence of viral hepatitis B among pregnant women at the CHUD-B is high. This best reflect this major public health problem due to Hepatitis B virus.

## Introduction

Les hépatites virales sont une cause majeure de morbidité et de mortalité, et elles sont classées comme la septième cause de décès dans le monde [1]. L'infection par le virus de l'hépatite B cause 70% de la mortalité aux hépatites [2]. L'Organisation Mondiale de la Santé (OMS) a estimé qu'en 2015, environ 240 millions de personnes étaient porteurs chroniques du virus de l'hépatite B (VHB) [3]. L'hépatite virale B est une maladie transmissible dont les modes de transmission sont bien connus: par le sang et ses dérivés, par voie sexuelle, la transmission verticale (de la mère à l'enfant). La transmission verticale a été identifiée comme une des causes de la prévalence élevée de l'infection par le virus de l'hépatite B en Afrique subsaharienne [4,5]. Chez les gestantes porteuses chroniques du VHB, contrairement à la population générale, de nombreux problèmes particuliers doivent être considérés, tels que l’influence de l’infection par le VHB sur la mère et le fœtus, l'influence de la grossesse sur la réplication du VHB, les effets du traitement antiviral sur la mère et le nouveau-né, la vaccination des nouveau-nés et la possible poussée d´hépatite après l'accouchement [6]. L'infection par le VHB, lorsqu'elle survient pendant la petite enfance est plus susceptible d'évoluer vers la chronicité [7,8]. Il apparait donc opportun de susciter une action en amont chez les femmes enceintes afin de limiter le risque de transmission verticale du VHB. C'est pour cela qu'il est recommandé de dépister toute femme enceinte vue en consultation prénatale au premier trimestre de la grossesse. Lorsque le diagnostic est fait, cela permet d'adopter des mesures prophylactiques basées sur la vaccination du nouveau-né contre l'hépatite B et l'administration d'immunoglobuline pendant les douze premières heures de vie [9,10]. La République du Bénin fait partie de la zone de forte endémie pour l'hépatite virale B [11]. C'est pour cela que cette étude s'est fixée pour objectif de déterminer la séroprévalence et les facteurs associés à l'hépatite virale B chez les gestantes de la région septentrionale du pays.

## Méthodes

**Patientes**: il s'est agi d'une étude transversale à visée descriptive et analytique avec recueil prospectif des données. Elle s'est déroulée du 1^er^ avril 2017 au 30 juin 2017. Cette étude a porté sur toutes les gestantes suivies ou venues accoucher dans le Service de Gynécologie-Obstétrique du CHUD-B de Parakou durant la période d'étude. Etait incluse, toute gestante ayant fait au moins une consultation prénatale et/ou ayant accouché dans le service pendant la période d'étude. Les gestantes ayant refusé de participer à l'étude ont pas été exclues. Les variables étudiées étaient l'AgHBs (variable dépendante) et les données socioéconomiques des gestantes (variables indépendantes).

**Technique d'échantillonnage**: la taille minimale de notre échantillon a été déterminée par la formule de Schwartz: N = t^2^p*(1-p)/m^2^ p=0,11 (séroprévalence de l'hépatite virale B chez les gestantes au Soudan du Sud selon une étude réalisée par Laku Stephen Kirbak *et al*. [12]); t=niveau de confiance (la valeur type du niveau de confiance de 95% sera 1,96) d'où t=1,96; m=marge d'erreur fixée à 5%; n=151 sujets. Un recrutement exhaustif de toutes les gestantes reçues en consultation prénatale ou aux urgences du service de gynécologie-obstétrique du CHUD-B a été effectué.

**Dépistage**: la recherche de l'AgHBs du VHB sur les prélèvements sanguins des gestantes a été faite par un test biologique de diagnostic rapide. Les résultats positifs ont été confirmés par la technique ELISA.

**Considérations éthiques**: les gestantes ont été incluses après consentement verbal libre et éclairé. Le respect de l'anonymat, de l'intimité et de la confidentialité des données a été observé. Par ailleurs, les femmes dépistées positives pour l'hépatite virale B ont été rassurées et orientées en consultation d'hépato-gastroentérologie pour le suivi et la prise en charge. Elles ont été sensibilisées sur l'importance de la vaccination contre le VHB de leurs nouveau-nés dès la naissance. Quant à celles qui ont été dépistées négatives, elles ont été orientées pour la vaccination contre l'hépatite B et ont été également sensibilisées sur l'importance de la vaccination contre le VHB de leurs nouveau-nés dès la naissance.

**Analyse des données**: les données recueillies ont été saisies avec le logiciel EPI Data version 3.5.1, puis traitées, analysées à l'aide du logiciel Epi-Info 7.1.14. En analyse univariée, les résultats des variables quantitatives ont été présentés sous forme de moyennes, avec leur écart type en cas de distribution normale. Les variables qualitatives ont été exprimées en effectif et pourcentage. En fonction des effectifs théoriques, nous avons procédé à l´usage du test de Chi carré de Pearson ou du test exact de Fisher. L'association entre la variable dépendante et chaque variable indépendante a été étudiée en réalisant le test du Chi carré ou le test exact de Fischer suivi de la p-value. Le seuil de significativité retenu a été de 5%.

## Résultats

**Description des gestantes enquêtées**: deux cent quatorze (214) gestantes ont été incluses. Leur âge moyen était de 26,73 ans ± 5,68. Parmi ces gestantes, 134 (62,62%) étaient mariées dont 98 (73,14%) selon le régime monogamique. Certaines gestantes (62 soit 28,97%) n'étaient pas instruites tandis que d'autres (113 soit 52,81%) avaient au moins un niveau secondaire. Parmi les 214 gestantes, 130 (64,49%) avaient un revenu mensuel inférieur au SMIG (40.000 FCFA). Il s'est agi de la première grossesse pour 64 gestantes (29,90%). L'antécédent personnel d'ictère a été retrouvé chez 31 femmes (14,58%). L'antécédent familial d'hépatite virale B a été rapporté par 9 gestantes (4,21%). L'âge médian de la grossesse pendant le dépistage était de 31 semaines d'aménorrhée (SA) et 126 femmes (58,88%) étaient au troisième trimestre de leur grossesse.

**Séroprévalence de l'hépatite virale B**: sur les 214 gestantes enquêtées, 30 femmes ont été dépistées positives à l'AgHBs soit une prévalence de 14,02%. À noter que tous les cas positifs au test de diagnostic rapide ont été confirmés par la méthode ELISA.

**Facteurs associés à la positivité de l'AgHBs chez les gestantes**: les facteurs statistiquement associés à la positivité de l'AgHBs chez ces gestantes sont: l'antécédent personnel d'ictère (p <0,001), l'existence de scarification (p=0,011) et l'antécédent familial d'hépatite virale B (p <0,001). Ces données sont consignées dans le Tableau 1. Les variables indépendantes suivantes ne sont pas statistiquement associées à la positivité de l'AgHBs chez ces gestantes: l'âge (p=0,909), la situation matrimoniale (p=0,880), le niveau d'instruction (p=0,749), le statut socio-professionnel (p=0,329), le revenu mensuel (p=0,160).

**Tableau 1:** facteurs associés à la positivité de l’AgHBs chez les gestantes enquêtées, Parakou, 2017

Facteurs associés	AgHBs (+) (n=30)		AgHBs (-) (n=184)		p-value
	Nombre	pourcentage	Nombre	pourcentage	
ATCD personnel d'ictère					
Oui	07	100	00	00	<0,0001
Non	23	11,11	184	88,89	
**ATCD familial d’HVB**					
Oui	06	66,67	03	33,33	<0,0001
Non	24	11,71	181	88,29	
**Scarifications**					
Oui	22	19,47	91	80,53	0,011
Non	08	07,92	93	92,08	

## Discussion

Dans le monde, 350 à 400 millions d'individus sont porteurs chroniques du VHB. L'Afrique du nord, avec une prévalence de 2 à 7%, est une zone d'endémicité intermédiaire tandis que l'Afrique subsaharienne est une zone de haute endémicité avec une prévalence comprise entre 8 et 18% de la population générale [11]. La république du Bénin faisant partie de l'Afrique subsaharienne, la prévalence de 14,02% que nous avons obtenue reste logique. Cette prévalence élevée témoigne de notre situation en zone géographique de forte endémicité et rend bien compte du problème majeur de santé publique que pose l'hépatite virale B dans notre pays. Ce chiffre est supérieur à celui retrouvé par Bigot *et al*. au cours d'une étude prospective réalisée en 1989 à Cotonou où la prévalence était de 8,26% [13]. Cette discordance de résultats dans ces deux études béninoises malgré la même méthodologie utilisée pourrait s'expliquer par la forte prévalence de l'hépatite virale B dans la région septentrionale du pays. En effet selon l'étude réalisée chez les nouveaux donneurs de sang en 2013 sur toute l'étendue du territoire béninois, la prévalence de l'infection par le VHB était de 20,15% dans le septentrion et de 9,08% dans les départements du Littoral et de l'Atlantique [14]. Notre résultat est similaire à ceux obtenus dans d'autres études notamment en Afrique subsaharienne. En effet, Candotti *et al*. [15] au Ghana en 2007 avaient retrouvé une prévalence de 12,2%. Au Soudan, cette prévalence était de 11% [12]. Sangaré *et al*. [16] à Ouagadougou en 2005, Sidibé *et al*. [17] à Bamako au Mali en 2001, Mamadou *et al*. [18] au Niger en 2012 avaient retrouvé des prévalences respectives de 11,4%, 15,5% et 16,6%. Par contre cette prévalence est nettement plus élevée que celles obtenues lors des études conduites en Afrique du Nord et en Europe. En effet, Hannachi *et al*. [19] en Tunisie en 2007, Çévik *et al*. [20] en Turquie retrouvaient des prévalences respectives de 4% et 4,2%. Cette différence observée pourrait être expliquée par l'épidémiologie locale propre à ces différentes régions.

Dans les pays, à forte endémicité pour l'hépatite B, le mode de transmission le plus fréquent est la transmission de la mère à l'enfant. Pour y remédier, il est recommandé de faire le dépistage des gestantes dès le premier trimestre de la grossesse [11]. Ou à n'importe quel moment même si elles ont été vaccinées avant de tomber enceinte [21, 22]. Dans notre étude, l'âge moyen des grossesses au moment du dépistage était de 31 semaines d'aménorrhée. Et plus de moitié des gestantes (58,88%) étaient au troisième trimestre de leur grossesse. Il revient donc aux sages-femmes et aux médecins gynécologues du CHUD-B, de réaliser ce dépistage à toutes les gestantes dès le premier trimestre de leur grossesse. L'AgHBs que nous avons utilisé dans notre étude pour le dépistage est un bon marqueur d'évaluation du portage du VHB dans une population puisque sa présence témoigne soit d'une hépatite virale B aigue, soit d'un état de portage chronique. Dans notre étude, l'âge de la gestante n'était pas lié à la positivité de l'AgHBs. Ce qui concorde bien avec les données épidémiologiques révélant la forte prévalence de la transmission verticale périnatale et horizontale du VHB sous nos cieux [13,23].

Dans notre série, le niveau d'instruction non plus n'était pas un facteur associé à la positivité de l'AgHBs. La prévalence de l'AgHBs variait très peu avec le niveau d'instruction. Un résultat similaire a été retrouvé dans l'étude réalisée par Angounda *et al*. [24] au Congo. En effet dans cette étude, la prévalence de l'AgHBs semblait diminuer avec le niveau d'instruction mais la différence observée n'était pas statistiquement significative. Notre étude a révélé une relation statistiquement significative entre la positivité de l'AgHBs et l'antécédent familial d'hépatite virale B. Le même constat est fait par Hannachi *et al*. [19] en Tunisie et Angounda *et al*. [24] à Brazzaville. Ces résultats corroborent les travaux antérieurs qui ont montré que l'acquisition de la maladie se faisait avant l'âge de 20 ans, plaidant en faveur d'une transmission verticale périnatale et horizontale intrafamiliale durant l'enfance et l'adolescence [25]. La transmission intrafamiliale à un jeune âge semble être l'un des modes de transmission les plus importants et un dépistage précoce de l'infection chez les gestantes permettrait la protection par vaccination de toutes les personnes vivant sous le même toit, ainsi que celle du partenaire. L'ictère est une manifestation présente dans un grand nombre de pathologies. Dans notre étude, toutes les gestantes séropositives à l'AgHBs avaient signalé un antécédent d'ictère révélant ainsi une relation statistiquement significative. Ceci pourrait s'expliquer par le fait que ces femmes avait fait une hépatite aigue symptomatique. Bani *et al*. [26] en Arabie saoudite avait abouti au même résultat en montrant une relation statistiquement positive entre la positivité de l'AgHBs et l'antécédent d'ictère. Il ressort également de notre étude que l'existence de scarifications était significativement associée au portage de l'AgHBs. Ce même constat a été fait par Sidibé *et al*. [17] au Mali en 2001 et Angounda *et al*. [24] au Congo-Brazzaville en 2014. Ce résultat peut s'expliquer par le fait que ces pratiques traditionnelles largement répandues dans notre société sont réalisées dans des conditions d'hygiène douteuses. Les effractions cutanées avec un matériel commun au cours des scarifications entrainent un risque de contact direct avec le sang contaminé favorisant ainsi la transmission du VHB.

## Conclusion

Cette étude a permis de déterminer la prévalence de l'antigène HBs ainsi que les facteurs de risque associés chez les gestantes au CHUD-B en 2017. Sur les 214 femmes enceintes incluses dans cette étude, 30 ont été dépistées positives à l'AgHBs soit une prévalence de 14,02%. Cette prévalence de l'AgHBs chez les gestantes au CHUD-B est élevée ce qui rend bien compte du problème majeur de santé publique que pose le VHB sous nos cieux. Dans notre étude, les facteurs associés à ce portage étaient la pratique des scarifications, l'antécédent personnel d'ictère et les antécédents familiaux d'hépatite virale B. La situation actuelle est préoccupante et des mesures urgentes dont le dépistage précoce des gestantes, doivent être prises pour réduire l'incidence de la maladie sous nos cieux.

### État des connaissances actuelles sur le sujet

- La séroprévalence de l'hépatite virale B chez les gestantes n'est pas actuellement connue dans la partie septentrionale du Bénin (dont Parakou);
- La transmission verticale (mère-enfant) contribuerait à entretenir cette prévalence élevée de l'hépatite virale B au Bénin.

### Contribution de notre étude à la connaissance

- La séroprévalence de l'hépatite virale B est connue et est élevée à Parakou;
- Les agents de santé, de ce fait, feront systématiquement le dépistage à toutes les gestantes vues en consultation;
- Des mesures préventives seront prises pour éviter la transmission verticale de la maladie.

## Conflits des intérêts

Les auteurs ne déclarent aucun conflit d'intérêts.

## References

[1] Stanaway JD, Flaxman AD, Naghavi M, Fitzmaurice C, Vos T, Abubakar I (2016). The global burden of viral hepatitis from 1990 to 2013: findings from the global burden of disease study 2013. Lancet.

[2] Bengsch B, Chang KM (2016). Evolution in our understanding of hepatitis B virus virology and immunology. Clin Liver Dis.

[3] World Health Organization (2015). Guidelines for the prevention, care and treatment of persons with chronic hepatitis B infection.

[4] Goldstein ST, Zhou F, Hadler SC, Bell BP, Mast EE, Margolis HS (2005). A mathematical model to estimate global hepatitis B disease burden and vaccination impact. Int J Epidemiol.

[5] Borchardt SM, Kocharian A, Hopfensperger D, Davis JP (2016). Prevention of perinatal transmission of hepatitis B virus: assessment among Wisconsin Maternity Hospitals. WMJ Off Publ State Med Soc Wis.

[6] Han GR, Xu CL, Zhao W, Yang YF (2012). Management of chronic hepatitis B in pregnancy. World J Gastroenterol.

[7] Pande C, Sarin S K, Patra S, Kumar A, Mishra S, Srivastava S (2013). Hepatitis B vaccination with or without hepatitis B immunoglobulin at birth to babies born of HBsAg-positive mothers prevents overt HBV transmission but may not prevent occult HBV infection in babies: a randomized controlled trial. J Viral Hepat.

[8] Li Z, Hou X, Cao G (2015). Is mother-to-infant transmission the most important factor for persistent HBV infection?. Emerg Microb Infect.

[9] Cheung KW, Seto MTY, Wong SF (2013). Towards complete eradication of hepatitis B infection from perinatal transmission: review of the mechanisms of in utero infection and the use of antiviral treatment during pregnancy. Eur J Obstet Gynecol Reprod Biol.

[10] Kim HY, Choi JY, Park CH, Jang JW, Kim CW, Bae SH (2013). Outcome after discontinuing antiviral agents during pregnancy in women infected with hepatitis B virus. J Clin Virol.

[11] Konaté Anselme (2012). Epidémiologie de l'infection par le virus de l'hépatite B en Afrique. Développement et santé.

[12] Laku Stephen Kirbak A, Nganga Z, Omolo J, Idris H, Usman A, Baguma Mbabazi W (2017). Sero-prevalence for Hepatitis B virus among pregnant women attending antenatal clinic in Juba Teaching Hospital, Republic of South Sudan. Pan Afr Med J.

[13] Bigot KA, Kodjoh N, Zohoun IS, Hountondji A, Latoundji S, Takpara L (1992). Séroprévalence de l'antigène HBs du virus de l'hépatite B chez les femmes enceintes et leurs enfants. Med Afr Noire.

[14] Kodjoh N (2015). Situation de la lutte contre les hépatites B et C en Afrique. Med Trop.

[15] Candotti D, Danso K, Allain JP (2007). Maternofetal transmission of hepatitis B virus genotype E in Ghana, west Africa. J Gen Virol.

[16] Sangaré L, Sombié R, Combasséré AW, Kouanda A, Kania D, Zerbo O (2009). Transmission anténatale du virus de l'hépatite B en zone de prévalence modérée du VIH, Ouagadougou, Burkina Faso. Bull Soc Pathol Ex.

[17] Sidibe S, Youssoufi Sacko B, Traoré I (2001). Prévalence des marqueurs sérologiques du virus de l'hépatite B chez les femmes enceintes dans le district de Bamako, Mali. Bull Soc Pathol Ex.

[18] Mamadou S, Ide M, Maazou ARA, Aoula B, Labo S, Bozari M (2012). HIV infection and hepatitis B seroprevalence among antenatal clinic attendees in Niger, West Africa. HIV AIDS (Auckl).

[19] Hannachi N, Bahri O, Mhalla S, Marzouk M, Sadraoui A, Belguith A (2009). Hépatite virale B chez les femmes enceintes tunisiennes: facteurs de risque et intérêt de l'étude de la réplication virale en cas d'antigène HBe négatif. Pathol Biol.

[20] Çevik E, Inan N, Baki Çentürk M, Demirel A, Kumbasar H, ArÜsoy A (2014). Seroprevalance of Hepatitis B among Pregnant Women and Neonates Born to HBsAg Positive Mothers in Batman. Viral Hepatitis J.

[21] Mast EE, Margolis HS, Fiore AE, Brink EW, Goldstein ST, Wang SA (2005). comprehensive immunization strategy to eliminate transmission of hepatitis B virus infection in the United States: recommendations of the Advisory Committee on Immunization Practices (ACIP) part 1: immunization of infants, children, and adolescents. MMWR Recomm Rep.

[22] US Centers for Disease Control (CDC) (1988). Prevention of perinatal transmission of hepatitis B virus: prenatal screening of all pregnant women for hepatitis B surface antigen. MMWR Morb Mortal Wkly Rep.

[23] Organisation Mondiale de la Santé (2017). Vaccins anti-hépatite B. Relevé épidémiologique hebdomadaire.

[24] Angounda BM, Bokilo A, Boumba LMA, Itoua C, Ahombo G, Moukassa D (2016). Prevalence of serologic markers and risk factors for Hepatitis B virus among pregnant women in Brazzaville, Congo. International Journal of Science and Research(IJSR).

[25] Mahadevan U, Kane S (2006). American gastroenterological association institute technical review on the use of gastrointestinal medications in pregnancy. Gastroenterol.

[26] Bani I, Mahfouz MS, Maki E, Gaffar A, Elhassan I, Yassin AO (2012). Prevalence and risk factors of Hepatitis B Virus among pregnant women in Jazan Region-Kingdom of Saudi Arabia. J Biol Agr Healthcare.

